# The impact of endometrial preparation for frozen embryo transfer on maternal and neonatal outcomes: a review

**DOI:** 10.1186/s12958-021-00869-z

**Published:** 2022-02-28

**Authors:** Jacqueline C. Lee, Martina L. Badell, Jennifer F. Kawwass

**Affiliations:** 1 Division of Reproductive Endocrinology and Infertility, Department of Obstetrics and Gynecology, Emory University School of Medicine, Emory Reproductive Center, 550 Peachtree Street, 18th Floor, Atlanta, GA 30308 USA; 2 Division of Maternal-Fetal Medicine, Department of Obstetrics and Gynecology, Emory University School of Medicine, Emory Perinatal Center, 550 Peachtree Street, 15th floor, Atlanta, GA 30308 USA

**Keywords:** Frozen-thawed embryo transfer, Endometrial preparation, Hypertensive disorders of pregnancy, Maternal outcome, Neonatal outcome, corpus luteum, Natural cycle

## Abstract

The use of frozen embryo transfer in assisted reproductive technology (ART) has steadily increased since development in the early 1980’s. While there are many benefits to delayed frozen embryo transfer, certain adverse perinatal outcomes are noted to be more common in these transfers when compared to fresh transfers, specifically hypertensive disorders of pregnancy. Frozen embryo transfers require coordination between the embryo’s developmental stage and the endometrial environment and can occur in either ovulatory or programmed cycles. Though there is no consensus on the ideal method of endometrial preparation prior to frozen embryo transfer, emerging data suggests differences in maternal and neonatal outcomes, specifically increased rates of hypertensive disorders of pregnancy in programmed cycles. Other reported differences include an increased risk of cesarean delivery, placenta accreta, postpartum hemorrhage, low birthweight, preterm birth, post term delivery, macrosomia, large for gestational age, and premature rupture of membranes in programmed cycles. The mechanism by which these differences exist could reflect inherent differences in groups selected for each type of endometrial preparation, the role of super physiologic hormone environments in programmed cycles, or the unique contributions of the corpus luteum in ovulatory cycles that are not present in programmed cycles. Given that existing studies are largely retrospective and have several key limitations, further investigation is needed. Confirmation of these findings has implications for current practice patterns and could enhance understanding of the mechanisms behind important adverse perinatal outcomes in those pursuing assisted reproduction.

## Background

Since the first infant was delivered after in vitro fertilization (IVF) in 1978, there has been a consistent increase in availability and utilization of assisted reproductive technology (ART). Simultaneously, developments and enhancements of many important laboratory technologies have transformed the field, including the introduction embryo cryopreservation in the early 1980s [1–3]. Globally, the frequency of frozen embryo transfers (FET) continues to increase, likely due to improvements in embryo survival with the introduction of vitrification, implementation of guidelines promoting single embryo transfer and therefore increased cryopreservation of supernumerary embryos, efforts to reduce rates of ovarian hyperstimulation syndrome (OHSS), utilization of preimplantation genetic testing, and the increase in embryo cryopreservation for fertility preservation [1, 4–8].

Despite rapid improvements in laboratory technology and clinical management, ART continues to be associated with an increased rate of perinatal complications when compared to natural conception [9, 10]. Since its inception, the field has been committed to maximizing patient and infant safety. Professional societies publish recommendations including guidelines limiting the number of embryos transferred based on age and embryo quality, in effort to reduce the rate of multiple gestation from ART [6, 11, 12]. Despite this, maternal and neonatal outcomes are still not equivalent to those of unassisted reproduction with reported increased rates of preterm birth, low birth weight, hypertensive disorders of pregnancy, cesarean delivery, and abnormal placentation, among others [9, 10, 13]. Inherent to the evaluation of safety in ART is the difficulty determining the contribution that underlying causes of infertility contribute to these outcomes versus potential risk from the ART procedures themselves.

Available data supports similar live birth rates after frozen transfers when compared to fresh transfers and even improved pregnancy outcomes in frozen transfers for certain groups [14–16]. When perinatal outcomes are compared, potential benefits of frozen transfers include lower risk of placenta previa, placental abruption, low birth weight, preterm birth, small for gestational age, and all cause perinatal mortality [17]. Conversely, frozen transfers have been associated with an increased risk of hypertensive disorders of pregnancy and increased rate of large for gestational age neonates [15, 17–22].

Suggested contributors to the observed increase in certain outcomes with frozen transfer include the effect of freezing and thawing of embryos before transfer or the influence of hormonal environment responsible for endometrial preparation prior to transfer. Notably, in many studies comparing perinatal outcomes in fresh and frozen transfers, the protocol by which the endometrium is prepared is not reported or considered in analysis. There is therefore growing interest in how the method of endometrial preparation may specifically influence outcomes in frozen embryo transfers.

## Main text

### Types of endometrial preparation

Endometrial preparation before frozen embryo transfer can occur in two types of cycles: ovulatory cycles and programmed cycles.

**Ovulatory cycles** rely, at least in part, on the development and activity of a dominant follicle, ovulation, and subsequent hormone production by the corpus luteum. Ovulatory cycles have several variations (Table 1). *Natural cycle* preparation occurs with no supplementation of hormones and ovulation occurs spontaneously after the midcycle surge of luteinizing hormone (LH). *Modified natural cycles* include the addition of a midcycle injection of human chorionic gonadotropin (hCG) to provoke ovulation and sometimes includes the addition of supplemental progesterone in the luteal phase. Finally in *stimulated cycles*, injectable gonadotropins, or oral medications such as letrozole or clomiphene citrate are used to induce ovulation of at least one follicle [23, 24]. In all ovulatory cycles, timing of transfer is based on ovulation, brought on either by LH surge or hCG administration, with consideration of the embryo’s developmental stage.

**Table 1:** Cycle characteristics for frozen embryo transfer preparation

	Estrogen	Timing of transfer	Progesterone
Ovulatory Cycles			
Natural cycles	Follicular development	LH surge	Corpus luteum
Modified natural cycles	Follicular development	hCG trigger	Corpus luteum with or without supplemental progesterone
Stimulated cycles	Follicular development	hCG trigger or LH surge	Corpus luteum with or without supplemental progesterone
Programmed Cycles			
Programmed cycles	Exogenous estrogen (oral, vaginal, transdermal)	Initiation of progesterone	Intramuscular and/or vaginal progesterone

**Programmed cycles** rely on the suppression of endogenous hormonal activity, often with GnRH agonist, and exogenous estrogen and progesterone are given to prepare the endometrium for the time of transfer [23]. Programmed cycles can also be called “artificial” or “hormone replacement” cycles throughout the literature.

There is no current consensus on the ideal endometrial preparation for frozen embryo transfer [24]. Two randomized control trials (RCT) comparing frozen embryo transfer cycles ovulatory cycles to those in programmed cycles showed similar results regarding key pregnancy outcomes including implantation rate, miscarriage rate and live birth rate [25, 26]. Another randomized controlled non-inferiority trial in the United Kingdom comparing modified natural versus programmed preparation for frozen embryo transfer confirmed lack of superiority of traditional programmed cycles when comparing clinical pregnancy, ongoing pregnancy, and live birth rate [27]. More recent studies have gone further to address maternal and neonatal outcomes between these two groups.

### Maternal and neonatal outcomes by method of endometrial preparation

The last several years has seen an influx of studies exploring differences in perinatal outcomes between different endometrial preparation techniques in frozen embryo transfer. Nine studies dedicated to this topic were selected by the authors based on knowledge of the existing literature and are outlined in this review. They are also summarized in Table 2.

**Table 2:** Summary of studies describing maternal and neonatal risks based on method of endometrial preparation for autologous frozen embryo transfer

Author (published)	Country	Design	Years	Method of cryopreservation	FET Preparation Groups	Maternal Significant Findings	Neonatal significant findings
von Versen-Höynck et al. (2019) [28]	United States of America	Prospective cohort study (single center)	2011–2017	Not listed	Modified natural cycle (*n* = 127) vs. programmed cycle (*n* = 94)	Programmed cycle vs. modified natural cycle: • ↑ Preeclampsia 12.8% vs. 3.9% (aOR 3.55; 95% CI 1.20–11.94) • ↑ Severe preeclampsia 9.6% vs. 0.8% (aOR 15.05; 95% CI 2.59–286.27).	Not studied
Jing et al. (2019) [29]	China	Retrospective Cohort study (single center)	2013–2016	Vitrification	Programmed cycle (*n* = 2611) vs. natural cycle with luteal progesterone (*n* = 8, 425)	Programmed cycle vs. natural cycle: • ↑ Hypertensive disorders of pregnancy 7.2% vs. 4.2% (aOR 1.780; 95% CI 1.262–2.510) • ↑ Cesarean section 85.9% vs. 78.4% (aOR 1.507; 95% CI 1.195–1.900)	None
Saito et al. (2019) [30]	Japan	Retrospective cohort study (multicenter)	2014	Not listed	Programmed cycle (*n* = 75,474) and natural cycle (*n* = 29,760)	Programmed cycle vs. natural cycle: • ↑ Cesarean section 44.5% vs. 33.7% (aOR 1.69; 95% CI 1.55–1.84) • ↑ Hypertensive disorders of pregnancy 4% vs. 3% (aOR 1.43; 95% CI 1.14–1.80) • ↑ Placenta accreta 0.9% vs. 0.1% (aOR 6.91; 95% CI 2.87–16.66) • ↓ Gestational diabetes mellitus 1.5% vs. 3.3% (aOR 0.52; 95% CI 0.40–0.68)	Programmed cycle vs. natural cycle: • ↑ Postterm delivery 0.9% vs. 0.3% (aOR 3.28; 95% CI 1.73–6.19) • ↑ Preterm delivery 8.8% vs. 7.4% (aOR 1.12; 95% CI 1.05–1.40)
Ginström Ernstad et al. (2019) [31]	Sweden	Retrospective cohort study (multicenter)	2005–2015	Vitrification and slow-freezing	Natural cycle (*n* = 6297) vs. stimulated cycle (*n* = 1983) vs. programmed cycle (*n* = 1446)	Programmed cycle vs. natural cycle: • ↑ Hypertensive disorders of pregnancy 10.5% vs. 6.1% (aOR 1.78; 95% CI 1.43–2.21) • ↑ Postpartum hemorrhage 19.4% vs. 7.9% (aOR 2.63; 95% CI 2.20–3.13 • ↑ Cesarean delivery 33.3% vs. 26.4% (aOR 1.39; 95% CI 1.21–1.60) Programmed cycle vs. stimulated cycle: • ↑ Hypertensive disorders of pregnancy 10.5% vs. 6.6% (aOR 1.61; 95% CI 1.22–2.10) • ↑ Postpartum hemorrhage 19.4% vs. 8.3% (aOR 2.87; 95% CI 2.29–3.60) • ↑ Cesarean delivery 33.3% vs. 28.9% (aOR 1.27; 95% CI 1.08–1.50)	Programmed cycle vs. natural cycle had increased risk of: • ↑ Post-term birth 8.9% vs. 5.8% (aOR 1.59; 95% CI 1.27–2.01) • ↑ Macrosomia 7.4% vs. 4.6% (aOR 1.62; 95% CI 1.26–2.09) Programmed cycle vs. stimulated cycle had increased risk of: • ↑ Post-term birth 8.9% vs. 4.7% (aOR 1.98; 95% CI 1.47–2.68) • ↑ Macrosomia 7.4% vs. 5.2% (aOR 1.40; 95% CI 1.03–1.90)
Wang et al. (2020) [32]	China	Retrospective cohort study (single center)	2014–2017	Vitrification	Natural cycle (*n* = 1947) vs. stimulated cycle (*n* = 1682) vs. programmed cycle (2333)	Not studied	Stimulated cycle vs. natural cycle: • ↓ Macrosomia 5.1% vs. 6.8% (aOR 0.74; 95% CI 0.57–0.97) Programmed cycle vs. natural cycle: • ↑ Large for gestational age 19.9% vs. 16.9% (aOR 1.25; 95% CI 1.05–1.49) Programmed cycle vs. stimulated cycle: • ↑ Large for gestational age 19.3% vs. 16.1% (aOR 1.25; 95% CI 1.08–1.46) • ↑ Macrosomia 7.8% vs. 5.7% (aOR 1.42; 95% CI 1.13–1.80)
Zong et al. (2020) [33]	China	Retrospective cohort study (single center)	2015–2018	Vitrification	Natural cycle (*n* = 4727) vs programmed cycle (*n* = 1642) and natural cycle vs. stimulated cycle (*n* = 517)	Programmed cycle vs. natural cycle: • ↑ Hypertensive disorders of pregnancy 7.9% vs 3.5% (aOR 2.00; 95% CI 1.54–2.60)	Programmed cycle vs. natural cycle: • ↑ Low birth weight 4.5% vs. 2.8% (aOR 1.49; 95%CI 1.09–2.06) • ↑ Preterm birth 7.9% vs. 4.6% (aOR 1.78; 95% CI 1.39–2.28) Stimulated cycle vs. natural cycle: • ↑ Preterm birth 7.7% vs. 4.6% (aOR 1.51; 95% CI 1.02–2.23)
Makhijani et al. (2020) [34]	United States of America	Retrospective cohort study (single center)	2013–2018	Vitrification	Natural cycle (*n* = 384) vs. programmed cycle (*n* = 391)	Programmed cycle vs. natural cycle: • ↑overall maternal complications 32.2% vs. 18.8% (aOR 2.21; 95% CI 1.51–3.22) • ↑hypertensive disorders of pregnancy 15.3% vs. 6.3% (aOR 2.39; 95% CI 1.37–4.17)	None
Zaat et al. (2021) [15]	Netherlands	Follow-up study to the ANTARCTICA randomized controlled trial (multicenter)	2009–2014	Vitrification and slow-freezing	Modified natural cycle (*n* = 45) and programmed cycle (*n* = 37)	Modified natural cycle vs. programmed cycle: • ↓ Hypertensive disorders of pregnancy in 6.7% vs 24.3% (RR 0.2;, 95% CI 0.08–0.94)	None
Hu et al. (2021) [35]	China	Retrospective cohort study (single center)	2013–2019	Vitrification	Natural cycle (*n* = 3790) vs. programmed cycle (*n* = 2561) and stimulated cycle (*n* = 670)	Programmed cycle vs. natural cycle: • ↑ Cesarean delivery 73% vs. 64% (aOR 1.52; 95% CI 1.35–1.71) • ↑ Hypertensive disorders of pregnancy 6% vs. 2% (aOR 2.84; 95% CI 2.11–3.83)	Programmed cycle vs. natural cycle: • ↑ Preterm delivery 12% vs. 8% (aOR 1.49; 95% CI 1.25–1.78) • ↑ Very Preterm delivery 2% vs. 1% (aOR 2.59; 95% CI 1.56–4.29) • ↑ Low birthweight 5% vs. 3% (aOR 1.75; 95% CI 1.34–2.28) • ↑ Macrosomia 13% vs. 10% (aOR 1.19; 95% CI 1.01–1.41) • ↑ Premature rupture of membranes 2% vs. 1% (aOR 1.67; 95% CI 1.12–2.49) Stimulated cycle vs. natural cycle: • ↑ Post-term delivery 0% vs. 0% (aOR 2.72; 95% CI 1.14–6.52) • ↑ Gestational diabetes mellitus 10% vs. 9% (aOR 1.64, 95% CI 1.28–2.11)

One of the first studies to report an association with hypertensive disorders of pregnancy with method of FET preparation was a prospective cohort study comparing outcomes in woman from the United States (U.S.) undergoing programmed FET and modified natural FET. The overall study stratified women by number of corpus luteum and included spontaneous pregnancies and fresh transfers, however when analysis was restricted to only FET cycles, pregnancies after programmed FET cycle were associated with higher risks for preeclampsia (12.8% vs. 3.9%, adjusted odds ratio (aOR) 3.55, 95% confidence interval (CI) 1.20–11.94) and preeclampsia with severe features (9.6% vs. 0.8%, aOR 15.05, 95% CI 2.59–286.27) compared with a modified natural-cycle FET after adjusting for the effect of age, nulliparity, history of hypertension, body mass index, polycystic ovary syndrome, and diabetes mellitus [28]. A parallel study also examined the role of the corpus luteum in maternal circulatory adaptations to pregnancy and will be addressed later in this review.

Shortly after, in 2019, two large retrospective studies were published comparing maternal and neonatal outcomes based on method of endometrial preparation [30, 31]. The first by Saito and colleagues used data from the Japanese Assisted Reproductive Technology Registry to compare outcomes in over 100,000 patients undergoing natural cycle FET and hormone replacement cycle, or programmed, FET. Of note, the natural cycle group included those where an hCG trigger or supplemental luteal progesterone were used. Pregnancies conceived in a hormone replacement cycle had an increased odds of hypertensive disorders of pregnancy (4% vs. 3%, aOR 1.43; 95% CI, 1.14–1.80], placenta accreta (0.9% vs 0.1%, aOR 6.91; 95% CI, 2.87–16.66) and cesarean section (44.5% vs. 33.7%, aOR 1.69; 95% CI, 1.55–1.84). Pre and post term delivery were also increased in hormone replacement cycles but there was a decreased odds of gestational diabetes mellitus (1.5% vs. 3.3%, aOR 0.52; 95% CI, 0.40–0.68) [30]. Authors considered maternal age, embryo stage at transfer, number of embryos transferred, use of assisted hatching and indication for ART in the adjusted analysis.

The second large retrospective study by Ginström Ernstad and colleagues used multiple Swedish registries to compare pregnancy outcomes in natural, stimulated, and programmed cycles where frozen embryos were transferred in over 8000 individuals between 2005 and 2015 that resulted in a singleton delivery. Using multivariable logistic regression controlling for potential confounding variables, authors found that pregnancies that occurred after FET in programmed cycles had a higher risk of hypertensive disorders in pregnancy (10.5 vs. 6.1%, aOR 1.78; 95% CI 1.43–2.21) and postpartum hemorrhage (19.4% vs. 7.9%, aOR 2.63; 95% CI, 2.20–3.13) when compared to natural cycles. Moreover, higher risks for post-term birth (8.9% vs. 5.8%, aOR 1.59; 95% CI, 1.27–2.01) and macrosomia (8.9% vs. 4.7%, aOR 1.62; 95% CI, 1.26–2.09) and cesarean delivery (33% vs. 26.4%, aOR 1.39; 95% CI, 1.21–1.60) were detected. When programmed cycles were compared to stimulated cycles these differences persisted.

Three additional single-center retrospective cohort studies from China explored maternal and neonatal outcomes based on cycle preparation [33, 35]. The first by Jing et al. showed increased rates of hypertensive disorders of pregnancy (7.2% vs. 4.2%, aOR 1.780; 95% CI, 1.262–2.510) and caesarean section (85% vs. 78.4%, aOR 1.507; 95% CI, 1.195–1.900) in programmed cycles and included pregnancies with multiple gestations [29]. The second by Zong and colleagues included only singleton deliveries found that pregnancies after programmed cycles had a higher risk of hypertensive disorders of pregnancy (7.9% vs. 3.5%, aOR 2.00; 95% CI 1.54–2.60), low birth weight (4.5% vs. 2.8%, aOR 1.49, 95% CI 1.09–2.06) and preterm birth (7.9% vs. 4.6%, aOR 1.78, 95% CI 1.39–2.28) when compared to natural cycle FET [33]. The risk of preterm birth was higher in stimulated cycles when compared to natural cycles with a confidence interval that approached one (7.7% vs. 4.6%, aOR 1.51, 95%CI 1.02–2.23).

The third and final study by Hu and colleagues included over 21,000 patients undergoing single frozen autologous blastocyst transfer [35]. Compared to natural cycle, programmed cycles had an increased risk of preterm delivery (12% vs. 8%, aOR 1.49, 95% CI 1.25–1.78), very preterm delivery (2% vs. 1%, aOR 2.59, 95% CI 1.56–4.29), and cesarean delivery (73% vs. 64%, aOR 1.52, 95% CI 1.35–1.71). Programmed cycles also had increased odds of low birthweight (5% vs. 3%, aOR 1.75, 95% CI 1.34–2.28), macrosomia (13% vs. 10%, aOR 1.19, 95% CI 1.01–1.41), premature rupture of membranes (2% vs, 1%, aOR 1.67, 95% CI 1.12–2.49) and hypertensive disorders of pregnancy (6% vs. 2%, aOR 2.84, 95% CI 2.11–3.83). Stimulated cycles were associated with an increased risk of preterm delivery and gestational diabetes mellitus when compared to natural cycle endometrial preparation.

In a study focused primarily on neonatal outcomes of singletons, Wang and colleagues showed differences in birthweight and gestational age at delivery when comparing natural cycle, simulated cycle and programmed cycle FET preparation on over nine thousand singletons conceived by autologous FET [32]. Authors used a propensity score matching method at a proportion of 1:1 to adjust for factors that influence the probability of receiving different FET cycle regimens. The stimulated cycle FET singletons had a lower adjusted odds of having macrosomia than the natural cycle FET singletons (5.1% vs. 6.8%, aOR 0.74; 95% CI, 0.57–0.97). Programmed cycle FET singletons had a higher adjusted odds of being large for gestational age than the matched natural cycle FET singletons (19.9% vs. 16.9%, aOR 1.25; 95% CI, 1.05–1.49) and matched stimulated cycle singletons (19.3% vs. 16.1%, aOR 1.25; 95% CI, 1.08–1.46). In the matched stimulated cycle FET and programmed cycle FET groups, the risk of large for gestational age neonates persisted and there was an increased odds of macrosomia in programmed cycles (7.8% vs. 5.8%, aOR 1.42; 95% CI 1.13–1.90).

Consistent with previous studies, a smaller retrospective cohort study based on a single U.S. clinic showed that programmed FET resulted in higher overall maternal complications (32.2% vs. 18.8%, aOR 2.21 95% CI 1.51–3.22), including higher probability of hypertensive disorders of pregnancy (15.3% vs. 6.3%, aOR 2.39; 95% CI 1.37–4.17). Analysis was limited to only singleton pregnancies from blastocyst transfers [34].

Finally, in one follow-up to an RCT, Zaat et al. showed that women who conceived by modified NC-FET have a decreased risk of hypertensive disorders of pregnancy compared with AC-FET (6.7% vs. 24.3%, relative risk 0.27; 95% CI 0.08–0.94; *P* = 0.031). Though the method of endometrial preparation was selected randomly, limiting confounders, only 82 women were included, and the study population was limited to only ovulatory women. Authors note that some outcome data was also collected retrospectively [15].

### Strengths and limitations

There are several strengths of the available studies observing differences in maternal and neonatal outcomes based on method of FET preparation. Many studies include large sample sizes with similar proportions of subjects having transfers in ovulatory and programmed cycles. Additionally, the two studies completed in Sweden and Japan used national used data from national registries which included an almost complete evaluation of individuals undergoing ART in their country of interest, limiting selection bias, and providing information on exposures and outcomes for the entire population [30, 31, 36]. Though the studies are heterogenous in outcome measures studied, in all studies that evaluated rates of hypertensive disorders of pregnancy in their analysis, programmed cycles had a higher risk of this diagnosis further suggesting but not confirming a causal relationship between programmed FET and hypertensive disorders of pregnancy [15, 28–31, 33–35].

Important limitations to the available data comparing maternal and neonatal outcomes across different endometrial preparation methods must be considered when deriving conclusions. In the available studies, the method of endometrial preparation is selected based on provider and patient preference or individual clinic policy, introducing the potential for significant differences between groups despite adjustments in analysis to account for potential underlying confounders. While programmed cycles share similar protocols across studies, the included and excluded ovulatory cycles vary from study to study (natural cycle, modified natural cycle, stimulated cycle). For example, modified natural cycle were considered “natural cycles” in the study by Saito et al. and categorized as “stimulated cycles” by Ginström Ernstad et al [30, 31].

.In addition, quantification of outcomes in these studies often relies on registry data or patient reported outcomes which can be limited by variation in coding or classification by those inputting data, missing data, and limited information about important confounders [36]. The investigations are heterogenous in outcomes reported and definitions used to categorize each outcome, and inclusion and exclusion criteria vary. Even hypertensive disorders of pregnancy, the most consistent outcome measure throughout the study, is considered as one group in some studies and stratified into different diagnoses (gestational hypertension, preeclampsia, etc.) in others. Extended time periods included in some analyses include embryos frozen by older slow freezing protocols or newer vitrification protocols and there is variation in number of embryos transferred and stage of embryo at time of transfer.

Most of the data available is retrospective and non-randomized and therefore subject to unrecognized confounding. The follow up study to the ANTARTICA randomized control trial by Zaat et al. is the only study where patients were randomized which limits confounding bias but is limited by small sample size and some data presented in this study was collected retrospectively. Moreover, odds ratios are generally < 2 and therefore may lack clinical significance [37]. Statistically significant findings reported in the current studies might have reflected the large sample sizes, selection bias and residual confounding rather than a true causal association.

Lastly, though many of the presented findings showed significant relative differences between endometrial preparation method as represented by adjusted odds ratios, the absolute differences in adverse events between protocol groups is often modest (Table 2). One must therefore interpret these results with caution given that small absolute differences noted between protocols are potentially of limited clinical importance to the individual patient and may not justify changes in clinical practice.

### Proposed mechanisms

There are three key proposed causes for the differences in the above maternal and neonatal outcomes reported. Although all studies attempt to control for possible confounders, the selection of endometrial preparation may be based on underlying factors that may contribute to differences in outcomes between the study groups. For example, if women in the hormone replacement group were more likely to have endocrine disturbances including hyperandrogenism, insulin resistance or dyslipidemia as seen in the study by Zong et al., this could drive the observed differences in pregnancy outcomes rather than role of endometrial preparation [33, 38, 39].

A second proposed difference is the difference in hormonal milieu, specifically the non-physiologic concentrations of estrogen and progesterone present in programmed cycles. Estrogen and progesterone play important regulatory roles in the development of the endometrial environment and establishment of early pregnancy [40]. It is therefore possible that supraphysiological concentrations of these hormones could interfere with normal development and the evolution of the clinical presentation of preeclampsia [41]. However, this does not explain the differences in hypertensive disorders of pregnancy seen between frozen and fresh transfers, as hormone levels are often well above physiologic levels in fresh transfers.

### The corpus luteum

The corpus luteum, a highly vascularized and heterogenous structure that forms from the follicular cyst after ovulation, plays an important role in the development of the secretory endometrium required for implantation and early pregnancy maintenance and has received significant attention for its role in frozen embryo transfer and preeclampsia pathogenesis in its absence [40]. Both progesterone and estradiol are secreted during the luteal phase correlating with episodic LH pulses. The corpus luteum also produces peptides such as oxytocin, relaxin, VEGF, inhibin, GnRH, growth factors and prostaglandins [40, 42]. When pregnancy occurs, hCG secreted from the trophoblasts stimulate the corpus luteum allowing for the continued secretion of substances that sustain and support early pregnancy.

Integral to a healthy pregnancy are appropriate maternal cardiovascular and renal adaptations. It has therefore been suggested that the differences in pregnancy outcomes seen in patients who pursue FET with a corpus luteum (ovulatory cycles) and without a corpus luteum (programmed cycles) are driven by the absence of substances, particularly the potent vasodilator relaxin, released by the corpus luteum in early pregnancy [43]. If there is deficient endometrial secretory development, altered trophoblast invasion and spiral artery remodeling, or attenuated systemic adaptation to pregnancy in programmed cycles, this could contribute to adverse pregnancy outcomes including hypertensive disorders of pregnancy [44].

Current studies assessing the differences in maternal cardiovascular and renal response in cycles with and without a corpus luteum further support this hypothesis. In one study investigating differences in relaxin concentration, creatinine as well as other maternal electrolyte concentrations showed that maternal serum creatinine, sodium, and CO2 were significantly higher in those with undetectable relaxin levels in conceptions with absent corpus luteum [45]. In the same study described above that looked at rates of preeclampsia based on number of corpus lutea, authors showed a blunted decline in the expected carotid-femoral pulse wave velocity and rise in carotid-femoral transit time most notable during the first trimester in patients without a corpus luteum when compared to those with one or with multiple [28]. When looking at several additional markers of vascular health in women with infertility, including those who underwent FET, there were several observed differences suggesting altered vascular health in women with absent or excessive (> 3) numbers of corpus lutea that could contribute to preeclampsia risk [46]. Furthermore, when compared to placentas from pregnancies with fresh transfers (corpus lutea present), those who underwent FET in a programmed cycle had more anatomic and vascular pathologies [47]. Placentas from ovulatory frozen embryo transfer pregnancies were not included but could be a source of future study.

### Future directions

Available data consistently identifies differences in rates of hypertensive disorders of pregnancy in programmed cycles [15, 28, 30, 31, 33–35]. Hypertensive disorders of pregnancy including gestational hypertension and preeclampsia, are pregnancy-specific hypertensive disorders that contribute significantly to maternal and perinatal mortality worldwide [48]. Risk factors include multifetal gestation, nulliparity, chronic hypertension and diabetes, obesity, maternal age over 35 and pregnancy from assisted reproduction, among others. Though incompletely understood, proposed pathophysiologic mechanisms include abnormal placentation, impaired angiogenesis leading to increased resistance at the uteroplacental interface contributing to downstream endothelial dysfunction. Other immunologic, inflammatory, environmental, or genetic factors likely also contribute to a variety of vascular, hematologic, hepatic, and renal changes seen in the spectrum of disease [48].

The window of implantation and early development of pregnancy are key areas that could play a role in the development of this spectrum of conditions. Diagnosis during pregnancy is also associated with long term cardiovascular disease risk, and prevention strategies are limited and include secondary prevention with aspirin for patients with identified risk factors [49, 50]. More knowledge regarding differences in the environment in which a pregnancy is established in the ART population have implications for the general obstetric population as well. Identifying modifiable risk factors, including method FET preparation, are crucial to reducing the burden of disease in ART conceived persons.

While hypertensive disorders of pregnancy are reported to be significantly increased in programmed cycles consistently, other key outcomes studied include increased cesarean delivery rate increased in four of the six studies where it was addressed (Table 2). Cesarean delivery increases the risk for severe maternal morbidity and has implications for a patient’s future reproductive health [51]. Reducing the rate of unnecessary cesarean delivery is one step toward improving maternal safety [52]. Additional reported difference includes significant an increase in extremes of fetal growth, disorders of placentation including placenta accreta and preterm delivery in programmed cycles. Each of these proposed differences has important implications for short and long term maternal and child health and warrant further investigation.

Currently, the method of endometrial preparation is chosen based on provider preference or in line with clinic policy with consideration for the patient’s underlying diagnosis. While programmed cycles allow for greater control over monitoring visits and transfer timing, transfers in natural cycles provide less flexibility, more frequent and less predictable ultrasound and hormone monitoring [42]. Clinics that primarily use programmed cycles to time transfer would need to make the necessary adjustments, including to laboratory workflow and provider availability, to adequately adapt to this type of preparation if supported by future studies. Method of frozen embryo transfer preparation may also become important variables in national surveillance systems to track outcomes prospectively.

Even if future data supports embryo transfer in ovulatory cycles, some patients will remain ineligible for this type of transfer, including patients with premature ovarian insufficiency, postmenopausal patients, and those without ovarian tissue either due to iatrogenic or congenital causes. As we learn more about the differences between programmed and ovulatory cycles, including the potential role of the corpus luteum and its secretions, it is possible that the programmed cycle could be further enhanced for these populations to help mitigate potential increased risks [53].

The ideal study required to determine the best method of endometrial preparation is a prospective multi-center randomized control trial with standard endometrial preparation protocols and definitions across all sites. Clinically relevant outcomes measured have consistent definitions determined in collaboration with obstetric and pediatric providers. Furthermore, attention must be given to the comparison of different types of ovulatory FET preparations, again with standard protocols, to understand the potential contribution of ovulation induction, hCG trigger and luteal progesterone supplementation. Clinical trials such as the “Impact of Corpus Luteum Presence or Absence in the Incidence of Preeclampsia After Frozen Embryo Transfer” (NCT04092829) and “Natural Versus Programmed Frozen Embryo Transfer (NatPro)” (NCT04551807) will offer promising insight into these important questions.

## Conclusions

Though there is not yet a consensus on the ideal method of endometrial preparation prior to frozen embryo transfer, emerging, largely retrospective data suggests differences in maternal and neonatal outcomes, specifically increased rates of hypertensive disorders of pregnancy in programmed cycles. The mechanism by which these differences exist could reflect inherent differences in groups selected for each type of endometrial preparation, the role of super physiologic hormone environments in programmed cycles, or the unique secretions from the corpus luteum in ovulatory cycles that are not present in programmed cycles. Available studies targeting this question have several key limitations, therefore prospective, randomized control trials are needed to explore these suggested differences. Interpretation of results must consider differences in both relative and absolute risk when determining if adjustments to clinical practice are indicated. Ultimately, confirmation of these findings has implications for current practice patterns and could supplement our understanding of the mechanisms behind important adverse perinatal outcomes and contribute to enhanced safety for those pursuing assisted reproduction.

## Data Availability

Data sharing is not applicable to this article as no datasets were generated or analyzed during the current study.

## Abbreviations

aOR: Adjusted odds ratio
ART: Assisted reproductive technology
CI: Confidence interval
FET: Frozen embryo transfer
hCG: Human chorionic gonadotropin
HDP: Hypertensive disorders of pregnancy
IVF: in Vitro Fertilization
LH: Luteinizing hormone
OHSS: Ovarian hyperstimulation syndrome
RCT: Randomized control trial
US: United States
